# A Peer-Led Electronic Mental Health Recovery App in an Adult Mental Health Service: Study Protocol for a Pilot Trial

**DOI:** 10.2196/resprot.8795

**Published:** 2017-12-07

**Authors:** Amelia Gulliver, Michelle Banfield, Julia Reynolds, Sarah Miller, Connie Galati, Alyssa R Morse

**Affiliations:** ^1^ Centre for Mental Health Research Research School of Population Health Australian National University Canberra Australia; ^2^ ACT Health Canberra Australia

**Keywords:** peer work, tablet app, study protocol, pilot trial, recovery, mental illness

## Abstract

**Background:**

There is growing demand for peer workers (people who use their own lived experience to support others in their recovery) to work alongside consumers to improve outcomes and recovery. Augmenting the workforce with peer workers has strong capacity to enhance mental health and recovery outcomes and make a positive contribution to the workforce within mental health systems and to the peer workers themselves. Technology-based applications are highly engaging and desirable methods of service delivery.

**Objective:**

This project is an exploratory proof-of-concept study, which aims to determine if a peer worker-led electronic mental (e-mental) health recovery program is a feasible, acceptable, and effective adjunct to usual treatment for people with moderate to severe mental illness.

**Methods:**

The study design comprises a recovery app intervention delivered by a peer worker to individual consumers at an adult mental health service. Evaluation measures will be conducted at post-intervention. To further inform the acceptability and feasibility of the model, consumers will be invited to participate in a focus group to discuss the program. The peer worker, peer supervisor, and key staff at the mental health service will also be individually interviewed to further evaluate the feasibility of the program within the health service and further inform its future development.

**Results:**

The program will be delivered over a period of approximately 4 months, commencing June 2017.

**Conclusions:**

If the peer worker-led recovery app is found to be feasible, acceptable, and effective, it could be used to improve recovery in mental health service consumers.

## Introduction

Peer work is a rapidly growing industry in Australia and internationally [1-4]. Current evidence suggests that peer work can make a positive difference in mental health services [1,5]; however, the role and definition of peer work is flexible and the practice is implemented in a variety of ways and settings [2]. Questions still remain as to how positive outcomes are best achieved and under what circumstances [5]. It is important to evaluate the feasibility of new peer work programs and to assess the acceptability of these programs for consumers, peer workers, and other staff.

Technology-based electronic mental (e-mental) health is a rapidly growing industry. These mental health interventions can be cost-effective to implement and there is growing evidence to support their effectiveness [6]. However, much more research is needed to understand how best to implement these tools in routine care [7]. Implementation challenges include low uptake and completion rates [8]. It has been proposed that peer support interventions may be able to increase the uptake and completion of e-mental health interventions [8]. The proposed project will assess the acceptability of presenting an e-mental health app led by a peer worker.

Peer support can be defined as “...a system of giving and receiving help founded on key principles of respect, shared responsibility, and mutual agreement of what is helpful” (page 1) [9]. Peer work can be paid or volunteer and workers use their lived experience of mental illness to inform their practice in providing emotional and social and instrumental support to other consumers [5,10]. Peer work is distinguished from other support work by several key factors, including (1) peer workers can instill hope through positive self-disclosure; (2) they can role-model skills for self-care and coping; and (3) they are able to develop a peer-to-peer relationship that is qualitatively different to a clinician-to-patient relationship [1]. The peer-to-peer relationship is characterized by trust, understanding, acceptance, and authentic empathy facilitated by shared or similar lived experiences [1,4,5].

Peers are found to be as effective or slightly more effective at producing desired outcomes, compared to usual staff, when employed in conventional roles in mental health services [1,11]. In these positions, peer workers have been found to increase the engagement of difficult to reach consumers, reduce rates of hospitalization and hospital stay duration, and decrease consumer substance use [1,5,12]. Peers can also be employed in diverse and flexibly defined peer specific roles [4]. There is evidence to suggest that in these circumstances peer workers can produce a range of benefits for consumers, particularly in regard to facilitating recovery. These benefits include an increased sense of independence and empowerment, improved self-esteem and confidence, improved social support and community integration, breaking down perceived stigma, and fostering a sense of hope through positive role modeling [1,5]. The current research protocol focuses on paid peer workers delivering a novel, technology-based recovery intervention in a public mental health care setting.

Peer work is not without challenges. The nature of peer support, particularly the requirement for self-disclosure, can make it difficult for peer workers to balance personal and professional boundaries in a workplace context [3-5,13]. In addition, the diverse and flexible definitions of peer work can lead to a lack of role clarity [13]. This can result in a range of workplace issues, including a lack of understanding and recognition of the value of peer work [3,4,13]. Effective training, appropriate management, professional supervision, and defined roles have been identified as key strategies for overcoming these challenges [3,13]. It is important to evaluate new peer work positions and programs to ensure the professional environment facilitates effective peer work and to identify areas that require improvement. In addition, reciprocity is a key element of peer support and peer workers have reported experiencing personal benefits from their occupation, including improved self-esteem and a sense of empowerment [4,5]. Through peer support training, voluntary work, and paid work, peers also have the opportunity to develop job skills and confidence [5,14,15]. Thus, peer work can provide people with a pathway to re-enter the workforce and facilitate a person’s continuing recovery. Due to the reciprocal nature of peer support, it is also important to determine whether peer-delivered services are acceptable for both consumers and peer workers.

### Aims

The aim of this project are to determine if a peer worker-led e-mental health recovery intervention is a feasible, acceptable, and effective adjunct to usual treatment for people with moderate to severe mental illness, as determined by the type of diagnosis, duration and/or intensity of symptoms and degree of functional impairment [16]. As an exploratory proof-of-concept study, the primary focus will be on elements of the program that consumers find useful or not useful, barriers and facilitators to implementation from the peer worker and service perspective, and primarily qualitative investigations of effectiveness for recovery outcomes. The findings of the research will help inform a larger investigation of the role of peer workers and e-mental health, which can guide the future implementation of these initiatives within existing mental health services.

### Objectives

The primary objectives of the study are (1) to investigate the feasibility and acceptability of embedding a peer worker in a public mental health service, from both the consumer and service perspectives; and (2) to assess the acceptability and effectiveness of a recovery-focused e-mental health program as an adjunct to treatment as usual for people with moderate to severe mental illness.

### Project Design

The exploratory project design involves the post-intervention evaluation of a single cohort of participants using the recovery-focused e-mental health program. To inform this proof-of-concept study, we will also conduct interviews with the peer worker and mental health service staff and a focus group to collect qualitative perspectives with participants. The study protocol, in accordance with the Standard Protocol Items: Recommendations for Interventional Trials (SPIRIT) checklist [17], is shown in Multimedia Appendix 1.

### Ethics Approval

The ethical aspects of this research were approved by the Australian Capital Territory (ACT) Health Human Research Ethics Committee (ETH.2.17.028) and The Australian National University Human Research Ethics Committee (ANU HREC 2017/338).

## Methods

### Researchers

Three of the researchers involved in the development and conduct of the study (AG, MB, ARM) have lived experiences of mental health problems and are currently working as consumer researchers. These experiences, together with a consumer and carer advisory group, offer unique insight into the development of the questionnaires and the evaluation of the study.

### Intervention

#### Peer Worker

A part-time (7 hours per week) peer worker with lived experience was recruited by ACT Health for the trial of a peer worker-led e-mental health program within its mental health service. The peer worker was required to have a qualification equivalent to a Certificate IV in Mental Health Peer Work, which is a professional course to develop specialist skills for working with clients facing mental health challenges [18]. The peer worker was required to (1) have direct personal lived experience of using mental health services; (2) have had a positive experience of recovery; and (3) have the ability and willingness to disclose personal experience of recovery in order to influence others positively. Their general duties were varied and included delivering programs, providing emotional support, developing trusting and professional relationships, and assisting other staff with the creation and review of recovery plans. The peer worker role statement is presented in Multimedia Appendix 2. The peer worker will be trained on the use and support of the Stay Strong app and will receive professional supervision and support for implementing the program by an experienced peer supervisor. However, they will not have experienced the recovery app as a client.

#### Stay Strong E-Mental Health App

The Stay Strong Care plan was developed and evaluated by Professor Tricia Nagel and developed into a mobile electronic app for iPads and other tablets in collaboration with Queensland University of Technology [19,20]. The app is a structured mental health and substance misuse intervention available through iTunes or Google Play. It is designed to be used as a collaborative tool between workers and service users and assists workers to guide service users through a structured, evidence-based mental health and substance use intervention.

Mental health workers may take on a range of roles when delivering e-mental health interventions, depending on the nature of the intervention and existing mental health skills of the worker [7]. Previous research with the Stay Strong app found that it may be most suitable for workers who are able to take on a coaching or therapist role [21]. In the current study, the peer worker will act in a coaching role and assist the person to complete the app and apply its concepts to their personal situation. We expect that the unique nature of the peer worker relationship, including the ability to provide hope for the future through role modeling, shared experience, and the capacity for assisting the person to disclose difficult personal issues [22] will facilitate and maximize the engagement of participants with the app content. Feedback from the peer worker will help inform the development of future protocols for the use of the app in mental health services.

Although the app was initially designed for use with Aboriginal and Torres Strait Islander service users and has some culturally-specific imagery and content [19], it has been approved by the authors for use in non-Aboriginal and Torres Strait Islander populations. It comprises a simple, highly visual design that does not rely on literacy and concentration levels. The Stay Strong tablet (iPad) app focuses on the consumer’s strengths and worries and helps them to set goals for change [23]. Consumers are first asked to identify the people in their life that help keep them strong, their relationships, and the role they play in the person’s life. They are then asked to identify their strengths in 4 areas of their life and this is represented visually as leaves on a tree. The more strengths they identify, the stronger and healthier the leaves become. Consumers also identify things in their life that take away their strength in the same 4 areas. The more worries that are input, the more the leaves on the tree wilt and turn yellow. This creates an interactive and visual representation of the parts in their life where they are strong and parts where they are not as strong. The app is designed for use by workers who have some training in mental health [19] but are not health professionals; its content focuses on goal setting and understanding strengths and vulnerabilities in relation to recovery.

### Eligibility Criteria

The mental health consumers who will be invited to participate are all existing consumers of an ACT Health community-based mental health service in Canberra, Australia. All consumers who have given informed consent will be eligible to participate in the study. Intended staff participants are the peer worker conducting the e-mental health program, the peer supervisor, and other staff of the mental health service.

### Recruitment

The e-mental health program will be delivered in 4 sessions to attendees at drop-in clinics during their usual appointments at the mental health service. It is anticipated that the program will be delivered over a period of approximately 4 months, commencing June 2017.

Recruitment into the e-mental health program will be conducted by staff at the mental health service. Attendees at drop-in clinics will be offered the opportunity to take part in a peer worker-led recovery program during their appointments at the clinic. This is partly to assess the utility of the app during a time in which the consumers must be present at the clinic to receive medication. The program will consist of completing the Stay Strong recovery app on iPads with the support of the peer worker over approximately 4 sessions. We initially conceptualized the peer worker to lead a group session for the app; however, group delivery is not feasible in the drop-in clinic setting, where consumers arrive and depart at varying times.

### Research Study

#### Evaluation Survey

After completion of the e-mental health recovery program, the consumer researchers will invite those who took part to participate in the evaluation survey. Participation will involve completion of a short questionnaire about experiences with the peer worker and the e-mental health program. We will also offer consumers the opportunity to take part in a focus group discussion to evaluate the program in more depth with researchers and each other. Participation in the recovery program and in the research for consumers will be handled separately—consumers will be offered the opportunity to take part in the program by the peer worker as part of their usual contact with the mental health service. The researchers will recruit consumers for the evaluation and it will be made clear to consumers that participation in the evaluation is completely voluntary and independent of their participation in the recovery program or the services they receive at the mental health service. Potential participants will also be informed that staff will not see their individual answers to minimize the chance of social desirability responses. The recruitment target for the research is 30 consumers across 3 clinics. Staff of the mental health service predicted this is a feasible target based on current attendance at the service.

#### Focus Groups

We will recruit up to 10 consumers who complete the questionnaire to participate in the focus group discussion(s). The focus group(s) will be described during the administration of the questionnaire. We will collect details for consumers who are interested in participating in the focus group and will follow-up with these consumers to schedule the focus group(s).

#### Staff Interviews

In addition to the consumers of the mental health service, staff involved in the trial of the peer worker-led e-mental health program will also be invited to participate in an interview. The peer worker will be interviewed about their experience of the delivery of the app. Supervisory and management staff at the service will not be involved in the delivery of the app, but will be asked to participate in one-on-one interviews to discuss their observations of the feasibility of embedding a peer worker and e-mental health program within the service from an operational point of view.

The evaluation materials for the survey, focus groups, and interview are presented in Multimedia Appendix 3.

### Outcomes

#### Consumer Questionnaire

We will measure the participants’ perception of their own recovery using the Self-Identified Stages of Recovery (SISR) measure [24]. The SISR is a single-item scale describing stages of recovery and the participants’ perception of the stage of recovery with which they currently identify. Participants are asked to indicate which of 5 statements most closely describes how they have been feeling over the past month, with higher ratings indicating more positive perceptions of recovery. Examples of statements are:

I don’t think people can recover from mental illness. I feel that my life is out of my control, and there is nothing I can do to help myself.

Question 1

I feel I am in control of my health and my life now. I am doing very well and the future looks bright

Question 5

The single-item of the SISR has been argued to measure a unique aspect of recovery not assessed by continuous measures [25]. In addition, the single-item SISR has demonstrated reliability and concurrent validity [26] as well as convergent validity for the staged model of recovery [25].

Seven questions designed by consumer researchers will subsequently ask participants to rate their experience with the peer worker and the program on a 4-point scale from “Not at all” to “Yes definitely.” The questions were designed to explore the acceptability of a peer worker and an e-mental health program as a part of the service, as well as investigate recovery and self-efficacy. There is an open-ended section for any further remarks. The questionnaire will be conducted at the conclusion of the final session of the program delivery, within the usual time that consumers are attending the clinic for treatment.

#### Focus Group

The focus group will follow a semi-structured protocol, investigating experiences with the Stay Strong program and its delivery. This will provide consumers with an opportunity to discuss and reflect on their experiences with the peer worker and the program in more detail than the rating scales allow. It will also enable identification of aspects of the program that consumers like and dislike to inform possible future delivery. The focus group method was selected to facilitate richer information where participants can share and build on each other’s ideas. However, we will consider an interview method if we cannot secure sufficient participants for a focus group. The focus group will also be scheduled during usual clinic hours in a meeting room at a mental health service clinic.

The focus group will be facilitated by one researcher with a second researcher as note taker/assistant. The group will be audio-recorded and participants will be advised in the information sheet and accompanying explanation that this is necessary to avoid missing anything and they should decline to participate if not comfortable with recording. They will also be advised that if they choose to withdraw during the focus group—it will not be possible to delete their contributions to that point.

#### Staff Interviews

**Figure 1:**
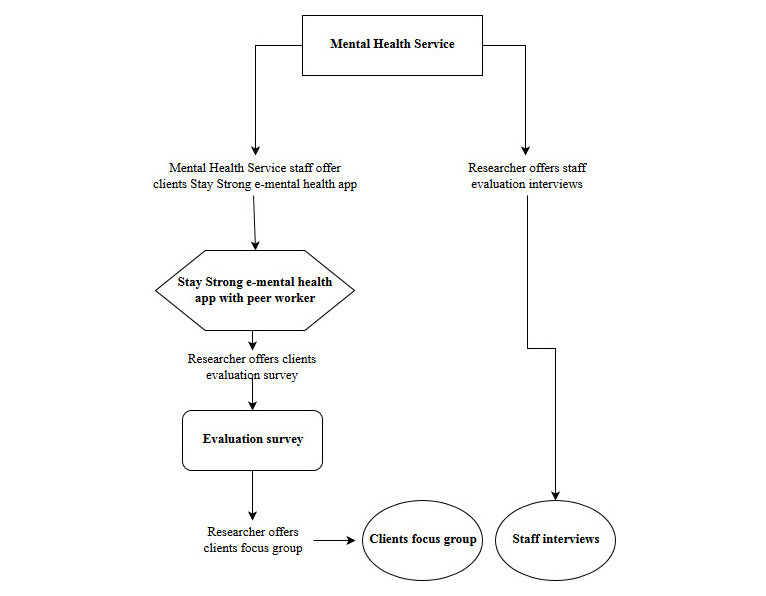
Recruitment flowchart.

Interviews will also follow a semi-structured protocol. The peer worker and other staff associated with the implementation of the program (eg, peer supervisor, health service coordinator) will be asked to discuss the benefits and challenges of the program, their experiences with both the peer worker model and the e-mental health program, and issues that may need to be considered for future delivery. Interviews with staff will be scheduled at a time and location convenient for the staff member. Interview participants will be asked if they consent to recording; if they refuse, the researcher will rely on written notes (Figure 1).

Questionnaires with consumers will be conducted across a period of approximately 4 months. The focus group and staff interviews will be conducted at the conclusion of the program. Data analysis and write-up is planned for completion by December 2017.

### Data Collection

All data collection will be conducted by members of the research team who will discuss protocols prior to commencing data collection to ensure consistency. Each participant will only be required to take part in the research on one occasion, with the exception of consumers who also choose to take part in the focus group. Due to the small sample and study location, no demographic data will be collected to minimize the chance of identifying individuals. All participants in the qualitative data collection will be advised that while every effort will be made to remove identifying information, they may still be identifiable from their comments.

### Data Management

All data will be stored securely in locked offices at The Australian National University (ANU). Consent forms and written questionnaires will be kept separately in locked filing cabinets. Digital data, including audio recordings, transcripts, and analysis files will be kept on password-protected computers at the ANU, accessible only to the research team. Identifying information will be removed from interview and focus group data during transcription and this will be reviewed by a member of the research team. Interview participants will have the opportunity to review their transcripts for accuracy and to identify any comments they do not wish to have included in publications. Data will be stored for a period of 5 years from last publication, after which it will be deleted and paper records destroyed.

### Analysis Strategy

Quantitative analyses of questionnaire responses will comprise descriptive statistics and will be conducted in SPSS (IBM) by 1 member of the research team. These will include mean SISR scores and mean ratings on each of the experience questions. Qualitative analyses of the focus group and interview transcripts will be managed using NVivo qualitative analysis software (QSR International). A framework analysis approach [15], incorporating both inductive and deductive coding, will be used to identify the key issues raised by consumers and staff. Initial coding will be conducted by 1 member of the research team and the coding frame discussed with the rest of the research team before coding is finalized.

### Risks and Benefits

The primary risk to consumers is that working through a recovery-oriented mental health program and answering questions related to recovery may raise uncomfortable memories or feelings, and may exacerbate mental health problems. However, the presence of a peer worker to support people as they work on the program, together with the specific purpose and format of the Stay Strong program are designed to minimize these risks. The study is being conducted as an adjunct to treatment as usual for this group, ensuring ongoing monitoring by mental health professionals.

It is expected that the people with mental health problems who participate in the study will increase their understanding of the psychosocial factors that influence their mental health, may develop skills in self-advocacy and self-support, and will develop a better understanding of the tools and skills they have to help them manage their well-being. They may also experience a sense of shared experiences and belonging with the peer worker and feel empowered by the opportunity to contribute to evaluating services.

The staff involved in the research will experience a different, non-clinical way of providing services to their consumers that may inform their own recovery-oriented practice. The standardized use of peer workers and e-mental health recovery interventions has the potential to improve both the availability of services (increased workforce) and to enhance self-help and maintenance of well-being in clinical care. This proof-of-concept study will provide preliminary data on the feasibility and acceptability of this approach.

### Quality Assurance and Monitoring

The design and progress of the study will be discussed with the Consumer and Carer Advisory Group for ACACIA: The ACT Consumer and Carer Mental Health Research Unit. This group comprises representatives from the ACT Mental Health Consumer Network, Carers ACT, ACT Health and independent consumers and carers from the community. The role of the advisory group is to provide feedback and assistance on ACACIA research projects, such as suggesting improvements to design and materials and assisting with recruitment.

Regular email and face-to-face meetings between the research team and the ACT Health team managing the program will also occur to monitor the progress of the program and the implications for the research study.

### Ethical Issues

The mental health consumers who will be invited to participate are all existing consumers of the mental health service and the intended staff participants are all employees of ACT Health. To minimize the risks presented by these existing relationships, recruitment of consumers into the e-mental health program will be undertaken by staff of ACT Health, whereas recruitment of consumers into the evaluation will be undertaken by a member of the research team employed by The Australian National University (ie, not an ACT Health employee and not a person involved in treatment). The information sheet details the principles of voluntary consent and participation, including that there will be no adverse consequences of choosing not to participate. The researcher present during the questionnaires, focus group(s), and interviews will verbally confirm understanding of the voluntary nature of participation before collecting written consent to proceed. Consumers can take part in the peer worker-led program and not be required to take part in its evaluation. In addition, the principles of voluntary participation for staff will also be discussed with the potential staff participants to ensure staff and their managers understand their rights to refuse to participate or withdraw without consequence.

### Informed Consent

All participants will be provided with a participant information sheet when invited to take part in the study. The key aspects of the study and ethical considerations will also be described verbally and participants will have the opportunity to discuss the study with researchers before agreeing to participate. In particular, the researchers will ensure that both consumers and staff understand that they are not obliged to participate in the research, regardless of their participation in the program and there are no consequences for refusing to take part. All participants will complete a written consent form (Multimedia Appendix 4). Consumers who choose to participate in the focus group in addition to the questionnaire will receive an additional information sheet explaining the issues particular to a group discussion and will sign a separate consent form.

## Results

### Dissemination

Results will be published in a report to ACT Health. A plain language summary will be provided to participants, to the consumer and carer organizations involved with ACACIA, and in ACACIA’s newsletter. A peer-reviewed publication will also be prepared and opportunities for conference presentations explored. Preparation of publications will be led by the principal investigator; authorship will be determined by contributions of each researcher to the entire process of conducting the project in accordance with publication guidelines. ACT Health partners will be offered the opportunity to contribute to the peer-reviewed publication and conference presentations. To minimize perceptions of conflict of interest, the research team will manage the research report and summaries for participants and the community.

### Funding and Declaration of Interests

Funding for the iPads to conduct the program and funding to employ the peer workers was provided by the Canberra Hospital Foundation and ACT Health, respectively. This funding applied to the e-mental health program delivery only.

The research study is being conducted using in-kind resources at the Centre for Mental Health Research. This includes funding provided by ACT Health for ACACIA: The ACT Consumer and Carer Mental Health Research Unit. The funding support for ACACIA is managed by a separate area from the one involved in program delivery and the research will be conducted independently of program implementation.

## Discussion

### Principal Findings

The current protocol describes an exploratory proof-of-concept study with results focused primarily on the feasibility of implementing the e-mental health program into service delivery models. We will also provide a post-intervention evaluation of the effectiveness of the program on recovery outcomes and the usefulness of the program from the consumers’ perspective. The study design is appropriate for the evaluation of an intervention and its feasibility in a real-world setting, using multiple perspectives and data collection methods. In addition, key strengths of the study include the consumer input into the evaluation design and the consideration of staff, peer worker, and consumer viewpoints in evaluating the intervention.

### Limitations

The study design does not allow clear separation of the concept of the peer worker and the app itself. However, participants will be explicitly asked to rate the aspects of the program separately to attempt to evaluate core aspects individually. Moreover, due to budget constraints, the study is utilizing a single peer worker, meaning that it will be difficult to differentiate opinions about the specific peer worker from the concept of a “peer worker”. We will attempt to ascertain this from the flexible questions in the participant focus group discussions and interviews with staff. In addition, we have acknowledged that there are challenges associated with peer work [3,13]; however, the purpose of this pilot study was not to overcome them per se. We have attempted to address these issues by providing specific training for the peer worker on the delivery of the app, evaluating the management and supervision of the peer worker (as indicated in the interview questions in Multimedia Appendix 3), and clearly defining the peer worker’s role in assisting the participants through the app.

### Conclusion

The findings from this study will have important implications for informing large-scale investigations into the role of peer workers using e-mental health, which can enable the development of guidelines to inform the future implementation of the provision of e-mental health using peer workers in mental health services.

## Appendix

Multimedia Appendix 1 Standard Protocol Items: Recommendations for Interventional Trials (SPIRIT) checklist.

Multimedia Appendix 2 Peer worker role statement.

Multimedia Appendix 3 Evaluation materials.

Multimedia Appendix 4 Model consent form for consumers.

## Abbreviations

ACACIA: The ACT Consumer and Carer Mental Health Research Unit
ACT: Australian Capital Territory
ANU: The Australian National University
e-mental: electronic mental
SISR: Self-Identified Stages of Recovery
