# Supplementary material for: A Peer-Led Electronic Mental Health Recovery App in an Adult Mental Health Service: Study Protocol for a Pilot Trial
Source: JMIR Res Protoc. 2017 Dec 7;6(12):e248. doi: 10.2196/resprot.8795 (PMC5740261; doi:10.2196/resprot.8795)
Supplement: Multimedia Appendix 2 [file resprot_v6i12e248_app2.pdf]

Multimedia Appendix 2. Peer Worker Role Statement

Overview of the work area and position:

The [mental health service] offer a multidisciplinary approach to the bio-psychosocial assessment and treatment of people with moderate to severe mental illness in a supportive and recovery oriented environment. The primary goal of the service is to optimise symptom relief, build capacity for self-management and resilience, and develop skills and resources for living in the community.

As a Peer Support Worker (PSW) you will be an integral member of the team and draw on your lived experience of recovery from mental illness in a positive and enlightening way to help and support others who are commencing or progressing through their own journey of recovery.

In working collaboratively you will be:

- Facilitating a peer group based on an e-health self-development program that focuses on individual strengths and values in aspiring towards personal goals and self-directed change; and
- Providing individual support to people focusing on their strengths to achieve identified goals.

Operational supervision and support will be provided by the Team Manager as well as by the multidisciplinary team of allied health professionals. Professional supervision will be provided by a senior peer support worker. The Centre for Mental Health Research will provide guidance in the use of the application/technology for the purpose of this pilot project.

Duties:

Under the supervision of an Allied Health professional, draw on your lived experience, knowledge, skills and abilities to:

1. Engage people and develop trusting and professional relationships that respect worker/person boundaries.
2. Seek to learn about the person's interests, their connections with family and friends, and work with the person in building their capacity to be part of their community.
3. Provide emotional support to people by constructively applying lessons learnt through your own lived experience, i.e. be a bearer of hope that recovery can and does occur.
4. Draw on your understanding and belief in strengths-based, recovery-oriented models of practice.
5. Provide opportunities to discuss problem solving strategies, reflecting on helpful and unhelpful behaviours.
6. Assist other staff members in the development and regular review of the person's individual recovery plan.
7. Work as part of a team in assisting people to engage with the practices associated with the collaborative recovery model, that is, assisting people in values and strengths, identification, and clarification, assisting the person to complete tasks as part of goal striving.
8. Use facilitation of the group program to engage with participants.

Participate fully as a team member by:

9. Cooperating closely with team members in order to ensure continuity of care and provision of a comprehensive service.
10. Actively participating in reflective practice through team meetings, decision-making processes, service planning sessions, supervision and staff development.
11. Contributing to the further development of best practice by informing policies and project submissions effectively drawing upon your personal experience of mental illness.
12. Consulting with people, contributing to regular evaluations of the effectiveness of the service.

Maintain records and resources by:

13. Collecting, collating and maintaining data on consumer contact.
14. With team members, collecting information on community resources.

Personal Attributes:

To be successful in this position, you will have a demonstrated track record of working in a multidisciplinary team environment and supporting and contributing to a healthy workplace that embraces diversity, encourages collaborative teamwork and complies with all the applicable regulatory and legislative requirements set out for such a role.

You will have a demonstrated:

- ability to communicate effectively, both verbally and non-verbally, in one-on-one situations including conflict resolution, negotiation and problem solving capability
- sound knowledge in relation to resources available in the community and be:
  - empathic
  - respectful
  - genuine
  - responsive
- understanding and knowledgeable of mental illness and its short and long-term effects and the adjustment process
- awareness of boundary issues in regard to the maintenance of a professional relationship and good organisational skills

It is expected that the successful candidate will have the following attributes and abilities:

- At a stage of their recovery where they are prepared to focus on the needs of others.
- Committed to non-judgmental support coupled with an understanding that people deal with their own situations differently
- Sensitive about a variety of issues faced by people, including psychological, adjusting to a disability, stress, grief and loss, financial, and relationship issues.

Peer support workers should inspire in others the hope and belief that recovery is possible.

Qualifications and experience:

Essential

i. Direct personal lived experience of using mental health services, with:

    - A positive experience of recovery; and
    - An ability and willingness to disclose personal experience of recovery in order to influence others positively.

ii. Certificate IV in Mental Health Peer Work

Desirable

- Experience, paid or voluntary, working in a mental health care setting
