# Supplementary material for: A Peer-Led Electronic Mental Health Recovery App in an Adult Mental Health Service: Study Protocol for a Pilot Trial
Source: JMIR Res Protoc. 2017 Dec 7;6(12):e248. doi: 10.2196/resprot.8795 (PMC5740261; doi:10.2196/resprot.8795)
Supplement: Multimedia Appendix 3 [file resprot_v6i12e248_app3.pdf]

Multimedia Appendix 3: Evaluation materials

1. Evaluation survey - participants

Self-Identified Stages of Recovery

People can feel differently about life with a serious illness at different times. Below are five statements about how people may feel.

Please read **all five statements** (A-E) and answer the question that follows.

A. "I don't think people can recover from mental illness. I feel that my life is out of my control, and there is nothing I can do to help myself." ☐

B. "I have just *recently* realised that people can recover from serious mental illness. I am *just starting* to think it may be possible for me to help myself." ☐

C. "I am *starting* to learn how I can overcome the illness. I've decided I'm going to start getting on with my life." ☐

D. "I can manage the illness reasonably well now. I am doing OK, and feel fairly positive about the future." ☐

E. "I feel I am in control of my health and my life now. I am doing very well and the future looks bright." ☐

Of the five statements above, which one would you say ***most closely*** describes how you have been feeling over the **past month** about life with the illness?

Tick the box next to that statement.

The peer worker-supported Stay Strong program

We would like to ask you a few questions about what you thought about the program and how it was presented to you:

1. Did you like that a **peer worker** was assisting you with the Stay Strong program?

No, not at all	No, not really	Yes, to some extent	Yes, definitely
☐	☐	☐	☐

2. Did you like completing the Stay Strong program in a **group**?

No, not at all	No, not really	Yes, to some extent	Yes, definitely
☐	☐	☐	☐

3. Did you like completing the Stay Strong program on the **iPad (electronic device)**?

No, not at all	No, not really	Yes, to some extent	Yes, definitely
☐	☐	☐	☐

4. Did you like completing the Stay Strong program during time that you **normally wait** around at the Health Service?

No, not at all	No, not really	Yes, to some extent	Yes, definitely
☐	☐	☐	☐

5. Did your participation in the Stay Strong program give you a **sense of control over your life**?

No, not at all	No, not really	Yes, to some extent	Yes, definitely
☐	☐	☐	☐

6. Did the Stay Strong program help you **feel that you could recover**?

No, not at all	No, not really	Yes, to some extent	Yes, definitely
☐	☐	☐	☐

7. Did the Stay Strong program help you feel **confident about your ability to take care of yourself**?

No, not at all	No, not really	Yes, to some extent	Yes, definitely
☐	☐	☐	☐

8. Please add any further comments about the program?

____

____

____

____

____

____

____

____

____

____

2. Focus group questions - participants

Questions:

1. What did you think about the peer worker and their involvement in assisting you with the Stay Strong program?

2. Did you notice any ways in which a peer worker ran the group differently to how a health professional would run a group?

3. This group explored one way that peer workers might help recovery in MH ACT. Can you suggest any other ways that they might contribute to the services offered by MHACT?

4. What was useful or what did you like about the Stay Strong program?

5. What did you dislike about the Stay Strong program?

6. What did you think about completing the Stay Strong program in a group?

5. What did you think about using an iPad to access the Stay Strong program?

7. What did you think about completing the Stay Strong program during time that you normally wait around at the Health Service?

8. Any other comments to add?

3. Interview questions – health professional

Questions:

We would like to ask you a few questions about your perceptions of delivering the program at the health service:

1. Were there any observable benefits for the health service associated with the delivery of the program? If yes, what were some of the benefits you observed?

2. Were there any observable disadvantages for the health service associated with the delivery of the program? If yes, what were some of the disadvantages you observed?

3. Were there any issues with the organisation of the program or groups? If yes, please tell us more?

4. What was your experience with organising the peer worker?

5. Do you feel that participants enjoyed working with the peer worker?

6. What did you like or find useful in the delivery of the Stay Strong program?

7. What did you dislike or find unhelpful in the delivery of the Stay Strong program?

8. Were there any issues or difficulties delivering the Stay Strong program in a group setting?

9. Were there any issues or difficulties delivering the Stay Strong program using the iPads?

10. Were there any issues or difficulties delivering the Stay Strong program using “dead time” i.e., time that would participants would have just spent waiting around?

11. Did you enjoy delivering the Stay Strong program at the health service?

12. Do you have any other comments about the program?

3. Interview questions – peer worker

Questions:

We would like to ask you a few questions about your perceptions of delivering the program:

1. Do you feel that participants enjoyed working with you specifically as a peer worker? Why or why not?

2. What did you like or find useful in the delivery of the Stay Strong program?

3. What did you dislike or find unhelpful in the delivery of the Stay Strong program?

4. What did you think about delivering the Stay Strong program in a group setting? Were there any benefits or drawbacks associated with this?

5. What did you think about delivering the Stay Strong program using the iPads?

6. Were there any issues or difficulties delivering the Stay Strong program using "dead time" i.e., time that would participants would have just spent waiting around?

7. Did you enjoy delivering the Stay Strong program at the health service?

8. Do you believe that participants were satisfied with what the Stay Strong program provided?

9. Do you have any other comments about the program?
