# Supplementary material for: A Peer-Led Electronic Mental Health Recovery App in an Adult Mental Health Service: Study Protocol for a Pilot Trial
Source: JMIR Res Protoc. 2017 Dec 7;6(12):e248. doi: 10.2196/resprot.8795 (PMC5740261; doi:10.2196/resprot.8795)
Supplement: Multimedia Appendix 4 [file resprot_v6i12e248_app4.pdf]

Principal Investigator
Dr Michelle Banfield
Centre for Mental Health Research
The Australian National University
Phone: 02 6125 6547
Email: Michelle.Banfield@anu.edu.au

Consent Form to Participate in a Research Project

I, ______________________________________ *(name of participant)*

have been asked to consent to my participation in a research project entitled:

Evaluation of a peer-worker supported e-mental health recovery program

In relation to this study I have read the Participant Information Sheet and have been informed of the following points:

1. Approval has been given by the ACT Health Human Research Ethics Committee.
2. The aim of the study is to **explore people's experiences of the peer-worker supported "Stay Strong" program.**
3. The results obtained from the study may or may not be of direct benefit to my mental health management.
4. The study procedure will involve **filling in a 10-15 minute survey**
5. Possible adverse effects or risks related to this study may include **mild feeling of emotional upset or discomfort**
6. Should I experience emotional upset or distress after participating in this study, I am aware that I may contact **my regular MHACT clinician, my General Practitioner or a Crisis Support Line**
7. Should I have any problems or queries about the way in which the study was conducted, and I do not feel comfortable contacting the research staff, I am aware that I may contact the ACT Health Human Research Ethics Committee Secretariat, Canberra Hospital, Yamba Drive, Garran ACT 2605 (ph: 6174 7968)
8. I can refuse to take part in this project or withdraw from it at any time before the survey is submitted without affecting my mental health care.
9. I understand that while the results of the research will be made accessible my involvement and my identity will not be revealed.

After considering all these points, I accept the invitation to participate in this study.

Name: (please print) ___________________________**Date:** ______________

Signature (Participant) _________________________
